# Predicting outcome in abdominal sepsis: putting the puzzle together

**DOI:** 10.1002/jcsm.12779

**Published:** 2021-08-19

**Authors:** Catherine S. Reid, Vanessa M. Banz, Joerg C. Schefold, Markus M. Luedi

**Affiliations:** ^1^ Department of Anaesthesiology, Inselspital Bern University Hospital, University of Bern Bern Switzerland; ^2^ Department of Visceral Surgery and Medicine, Inselspital Bern University Hospital, University of Bern Bern Switzerland; ^3^ Department of Intensive Care Medicine, Inselspital Bern University Hospital, University of Bern Bern Switzerland

Acquired muscular weakness and muscle wasting are frequently observed in critically ill patients, especially after a prolonged time in the intensive care unit (ICU).^1^ Patients with severe pulmonary and/or abdominal infections are at increased risk for *intensive care unit‐acquired weakness* (ICUAW), which may present as *critical illness myopathy*, *critical illness polyneuropathy*, and/or their combination—*critical illness polyneuromyopathy*.^2^, ^3^ Both ICUAW and muscle wasting are serious medical conditions, observed in up to 40% of respective patients.^1^, ^4^ Current data indicate that impaired weaning from mechanical ventilation and additional neuromuscular dysfunction are frequent in patients with severe abdominal infections.^5^ Despite aggressive medical treatment, patients suffering from abdominal sepsis regularly require prolonged treatment in the ICU; this can cause a vicious cycle of prolonged bed rest and/or need for sedation that contributes to pronounced ICUAW, unfavourable clinical outcomes, and mortality rates remained high for decades.^6^

More than two decades ago, single prediction scores such as the ‘Acute Physiology And Chronic Health Evaluation’ II or the ‘Mannheim Peritonitis Index’ were used to prognosticate clinical outcomes in patients with severe peritonitis and intra‐abdominal sepsis.^7^ More recently, we have learned that no single score can reliably predict outcomes in individuals suffering from peritonitis and that complex patient‐related criteria and treatment‐specific parameters should best define the outcomes of affected ICU patients. For example, Petersen *et al*. demonstrated in 2021 that outcome prediction in peritonitis significantly increases when multiple patient‐derived factors and treatment‐specific variables are integrated into a multidomain prediction model.^8^

In patients suffering from critical illness, acute muscle wasting often develops early (i.e. in the first few days of ICU treatment) and is accelerated in the presence of additional (multi‐) organ dysfunction.^9^, ^10^ Retrospective data indicate that decreased muscle mass is a predictor of increased in‐hospital mortality in elderly patients with sepsis.^11^ In this issue of the *Journal of Cachexia, Sarcopenia, and Muscle*, Cox *et al*. provide important prospective data about the impact of acute muscle mass loss and sarcopenia on long‐term outcome in critically ill patients with intra‐abdominal sepsis.^12^ The authors included 47 ICU patients suffering from intra‐abdominal sepsis and followed them up for 1 year. Abdominal computed tomography scans were performed to assess skeletal muscle index.^12^ This study confirmed that loss of muscle mass starts acutely and early in sepsis and persists over time, leading to a considerable delay in clinical recovery.^12^ The authors observed that pre‐existing sarcopenia, a condition which may be observed in a variety of chronic illnesses, is a predictor of poor functional outcome and increased mortality after 1 year.^12^ While this study focused exclusively on patients with intra‐abdominal sepsis, the results are in line with other studies evaluating sarcopenia, thus strengthening its value in predicting outcomes.^13^, ^14^

The predictive synthesis of patient‐related parameters and treatment‐specific criteria can be amplified by the integration of molecular findings.^15^ Inexpensive cell‐derived biomarkers used to detect bacterial infection—such as the delta‐haemoglobin equivalent and granularity index—are readily available and easily measured in a standard laboratory.^16^ These simple lab tests, together with a multidomain prediction model and CT scans for skeletal muscle index measurement, could be integrated into a diagnostic/predictive arsenal, alerting clinicians to which peritonitis patients are at higher risk for adverse outcomes. While the acute loss of muscle mass in patients suffering from abdominal sepsis may be difficult to prevent, maximal therapy could be tailored to individuals deemed ‘high risk’.^17^, ^18^

Cachexia and sarcopenia have already been established as risk factors for unfavourable clinical outcomes in chronic disease; we are however still gaining a better understanding of the relevance of acute muscle loss in acute abdominal sepsis. Cox *et al*. provide important additional prospective evidence for putting this particular puzzle together. Sarcopenia and muscle wasting are not just innocent bystanders in abdominal sepsis but play a significant role in determining patient outcome.

## Funding

No funding involved.

## Author contributions

Catherine S. Reid, Vanessa M. Banz, Joerg C. Schefold, and Markus M. Luedi helped in writing the article.

## Conflict of interest

Catherine S. Reid reports no conflicts of interest, Vanessa M. Banz reports no conflicts of interest, and Joerg C. Schefold reports grants unrelated to the submitted work from (full departmental disclosure) Orion Pharma, Abbott Nutrition International, B. Braun Medical AG, CSEM AG, Edwards Lifesciences Services GmbH, Kenta Biotech Ltd., Maquet Critical Care AB, Omnicare Clinical Research AG, Nestle, Pierre Fabre Pharma AG, Pfizer, Bard Medica S.A., Abbott AG, Anandic Medical Systems, Pan Gas AG Healthcare, Bracco, Hamilton Medical AG, Fresenius Kabi, Getinge Group Maquet AG, Dräger AG, Teleflex Medical GmbH, Glaxo Smith Kline, Merck Sharp and Dohme AG, Eli Lilly and Company, Baxter, Astellas, Astra Zeneca, CSL Behring, Novartis, Covidien, Philips Medical, Phagenesis Ltd., Prolong Pharmaceuticals, and Nycomed. The money was added to departmental funds. No personal financial gain applied. Markus M. Luedi reports no conflicts of interest.
